# Surgical versus percutaneous isolated pelvic perfusion (IPP) for advanced melanoma: comparison in terms of melphalan pharmacokinetic pelvic bio-availability

**DOI:** 10.1186/s13104-017-2738-y

**Published:** 2017-08-15

**Authors:** Stefano Guadagni, Giancarlo Palumbo, Giammaria Fiorentini, Marco Clementi, Luca Marsili, Aldo Victor Giordano, Francesco Masedu, Marco Valenti

**Affiliations:** 1 Department of Applied Clinical Sciences and Biotecnology, University of L’Aquila, via Vetoio, 67100 L’Aquila, Italy; 2 Department of Life, Health and Environmental Sciences, University of L’Aquila, via Vetoio, 67100 L’Aquila, Italy; 3 Medical Oncology Unit, Department of Oncology and Hematology, Azienda Ospedaliera “Ospedali Riuniti Marche Nord”, Pesaro, Italy

**Keywords:** Melphalan, Isolated pelvic perfusion, Melanoma

## Abstract

**Background:**

Isolated pelvic perfusion (IPP) can be used to treat unresectable melanoma metastases of the pelvis. IPP can be performed either by surgical or percutaneous approaches, using different balloon catheters. The aim of this study was to examine whether the surgical and percutaneous approaches were comparable with respect to tumor drug exposure in the pelvis.

**Methods:**

A pharmacokinetic study was performed in 5 melanoma patients treated with surgical IPP and five with percutaneous IPP. Both groups received melphalan at the dose of 30 mg/m^2^. Melphalan pharmacokinetic analyses were performed and the main parameter used to evaluate pelvic tumor drug-exposure was the ratio of areas under the melphalan plasma concentration curves in the pelvis and the systemic compartment, during the perfusion time (AUC_0 to 20_). Non-parametric Mann–Whitney tests were employed for statistical comparisons.

**Results:**

The median and interquartile range (IQR) values of the ratios between melphalan AUC_0 to 20_ in pelvic and systemic compartments were 7.9 (IQR 7.2 to 9.9) and 5 (IQR 4 to 7.9) for surgical and percutaneous IPPs, respectively (p = 0.209).

**Conclusions:**

Tumor exposure to drug using these two methods did not statistically differ and both methods, therefore, can be adopted interchangeably, utilizing a perfusion blood flow rate of approximately 120 ml/min. The small sample size is a limitation of this study but our preliminary results can be used to calculate the effect size of a larger trial.

*Trial Registration* Clinical Trials.gov Identifier NCT01920516; date of trial registration: August 6, 2013

**Electronic supplementary material:**

The online version of this article (doi:10.1186/s13104-017-2738-y) contains supplementary material, which is available to authorized users.

## Background

Regional chemotherapy is an option for relapsed patients with advanced cancers in the pelvic and groin areas. In the 1950s [1–3], surgeons developed isolated pelvic perfusion (IPP), with the purpose of isolating the pelvic circulation by blocking blood-flow in the aorta and inferior vena cava with balloon catheters and at the level of the thigh with pneumatic cuffs, with the pelvic area subsequently perfused with antineoplastic drugs via extracorporeal blood circulation. Indeed, several IPP techniques have been published [4] and a subsequent pharmacokinetic study demonstrated that there is drug leakage from the pelvic compartment [5]. Improvements in procedure to reduce morbidity and side-effects include a simplified technique, called hypoxic pelvic perfusion, based on the use of two balloon catheters positioned by femoral vessel exposure [6]. This technique is similar but simpler to that of Turk et al. [7], which involves the use of four catheters. Aigner’s technique [6] has now been fully evaluated by van Ijken et al. [8, 9] in both animals and humans, in terms of feasibility, pharmacokinetics, adverse events and clinical outcomes. Further significant improvements were reported using a percutaneous hypoxic IPP technique proposed by Thompson et al. [10]. This technique, characterized by the use of 18-French (Fr.) introducers [11], has been further improved by Ricci et al., using 11-Fr. introducers [12]. A percutaneous approach employing two cannula sheaths was reported by Bonvalot et al. [13], whereas Begossi et al. [4] and more recently Murata et al. [14] used four cannula sheaths (two 9-Fr. and two 6-Fr.). Hemofiltration, used at the end of IPP to reduce the drug-related side effects, has been shown to be a safe and effective procedure [5, 15, 16].

The surgical method that exposes femoral vessels is recommended when lymphadenectomy is required but is rarely repeatable due to the formation of scar tissue. The percutaneous approach is, therefore, more feasible in patients that require repeated IPPs to prolong clinical response and survival [14].

To date, no reports have compared surgical and percutaneous approaches with respect to pelvic tumor drug exposures. Therefore, we have tested the hypothesis that pelvic tumor drug exposure would be similar using different catheters under conditions of similar blood flow rates in a pharmacokinetic study performed on 10 patients with loco-regional melanoma metastases to pelvic and/or groin regions, employing hypoxic IPP delivering of Melphalan, with subsequent hemofiltration to reduce side-effects (Additional file 1).

## Methods

### Eligibility criteria

This interventional pilot study has been performed in accordance with the Declaration of Helsinki and has been approved by the ethics committee of University of L’Aquila, L’Aquila, Italy, as part of a larger study (ClinicalTrials.gov Identifier NCT01920516). Written informed consent was obtained from each of the 10 patients, presenting with loco-regional melanoma metastases to the pelvis and or inguinal region, after receiving complete information concerning their disease and the implications of the proposed palliative treatment, in accordance with the ethical standards of L’Aquila University committee on human experimentation.

Patients (Table 1) included in the study had stage IIIC melanoma [17], life expectancy >3 months, and a Karnofsky performance scale index of >60 [18]. Patients with metastases in the lower part of the thigh, extrapelvic metastases, renal and liver failure, deep venous thrombosis, severe atherosclerosis or coagulopathy, were excluded from this study. Before perfusion, all patients were subjected to angiography or angio-CT of the aorto-iliac tree and inferior vena cava. Limitations included: (1) no percutaneous perfusion when common femoral artery diameter was ≤7 mm, making vessel dissection risky; (2) when surgical procedures performed previously had resulted in femoral vessels fibrosis, exposure of iliac vessels was necessary. Demographic data (Table 1) was collected by the medical team during operative procedures and recovery.

**Table 1:** Sample timing

Sampling site	Sampling time (min)									
Pelvic (inferior cava vein)	5	7	14	17	20					
Systemic (periferal vein of the arm)	5	7	14	17	20	25	30	45	60	80
Blood exiting patients via extracorporeal circuit						25	30	45	60	80
Ultrafiltrate						25	30	45	60	80

### Hypoxic pelvic perfusion techniques

**Fig. 1:**
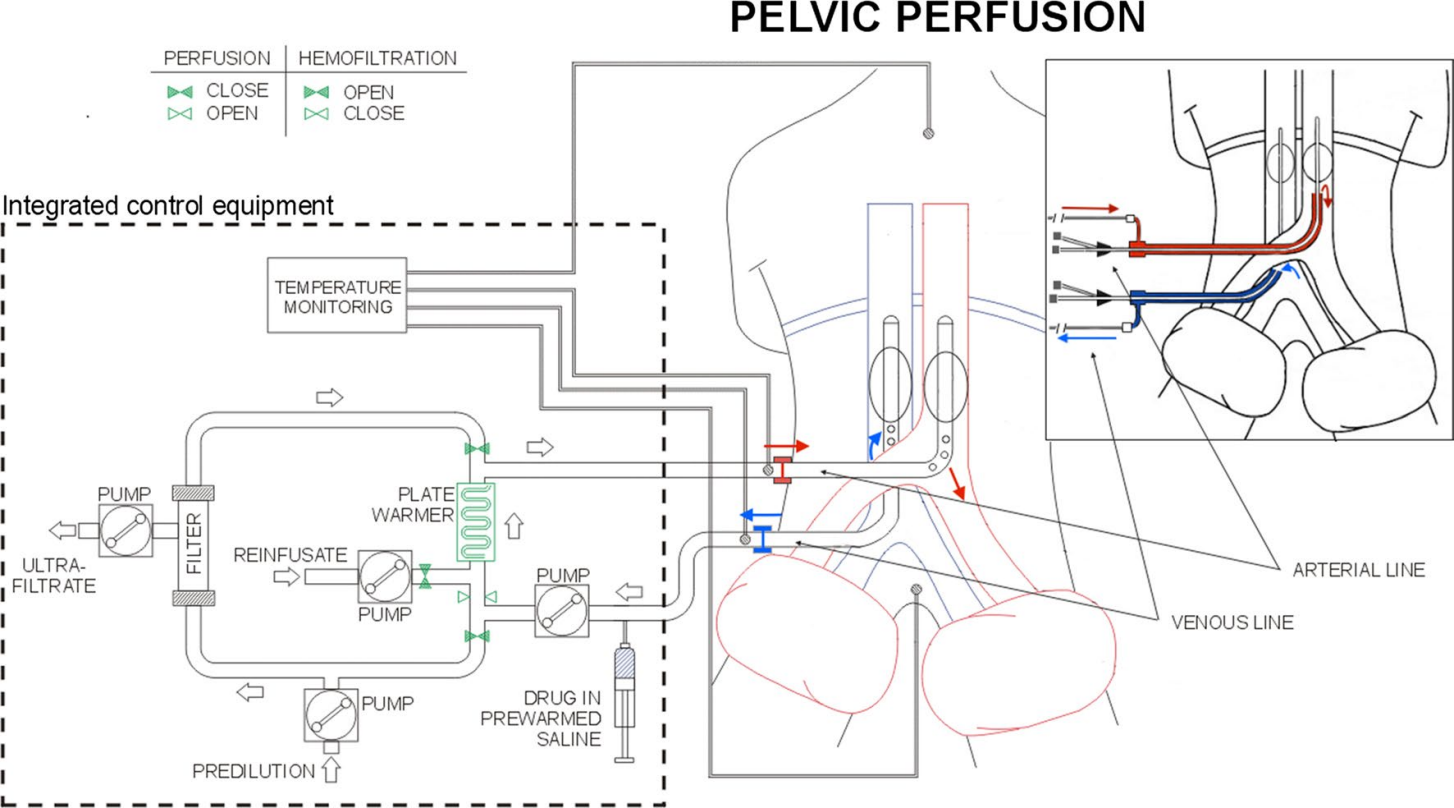
Schematic diagram of surgical and percutaneous (in cartouche) IPP with hemofiltration

Perfusions were performed under general anesthesia, as previously described [15]. Hypoxic perfusion with hemofiltration has three phases: the first, the isolation phase, is characterized by the blockage of blood flow to the aorta and inferior cava, using endovascular balloon catheters, at the level of the thighs by pneumatic cuffs and can be performed by both surgical or percutaneous methods (Fig. 1).

**Fig. 2:**
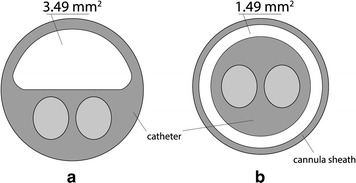
Cross sections of **a** 3-lumen, 12-Fr. balloon catheter; **b** 2-lumen, 8-Fr. balloon catheter and 11-Fr. cannula sheath introducer (the blood flows in the *white area*)

In the surgical approach, following systemic heparinization (150 U/kg heparin), a 3-lumen, 12-Fr. balloon catheter (pfm medical ag, Cologne, Germany) was introduced into the inferior vena cava via the saphenous vein (or iliac vein) and into the aorta via the femoral artery (or iliac artery). Catheters are positioned below renal vessels but above aortic and venous bifurcations, using a fluoroscopically guided wire. One of the three lumens of the catheter (Fig. 2a) is used for blood circulation and the other lumens used for inflating balloons and positioning the guide wire.

In the percutaneous procedure, femoral vessels were punctured using two 11-Fr. introducers, each with a hemostatic valve and dilatator (Radifocus; Terumo, Tokyo, Japan): (1) an arterial introducer of 25 cm length and (2) a venous introducer of 10 cm length, and 2 double-lumen 8-Fr. balloon catheters (pfm medical ag) were inserted, each with one lumen utilized to inflate the balloon and the other to position the guide wire. Blood circulation and drug perfusion was accomplished in the long, hollow cylindrical space between the introducer wall and the catheter, with blood flowing through a ring surface at the top of the space (Fig. 2b).

In order to suspend blood flow, balloons were inflated with radiopaque diatrizoate dye diluted in an isotonic sodium chloride solution. Immediately prior to the initiation of perfusion, in order to complete pelvic circulation isolation, two large-cuff orthopedic tourniquets were positioned around each thigh and inflated.

The second phase, the perfusion phase, is characterized by extracorporeal blood circulation. Infusion channels of arterial and venous surgical catheters or hemostatic valve channels of the 11-Fr. introducers were connected to a hypoxic perfusion tube set mounted on a regional, cancer therapy-dedicated, circulation device (Performer LRT; RanD, Medolla, Italy). The circuit was primed with an isotonic sodium chloride solution containing heparin (10,000 U/l). Drug perfusion was initiated upon establishing the required blood flow rate of approximately 100 ml/min in the extracorporeal circuit (aspiration from the inferior cava vein and infusion into the aorta). Melphalan (30 mg/m^2^), diluted in 250 ml of an isotonic sodium chloride solution containing 16 mg of dexamethasone sodium phosphate, was administered via the circuit over a 3-min period. The extracorporeal circuit (Fig. 1) connected to the circulation device contains a heating element and a hemofiltration module, both of which were controlled by the device during perfusion and subsequent hemofiltration phases and perfusion was continued for 20 min. Temperature loss approximated to 1 °C/m of tubing and the length of the tubing was 5 m. Therefore, to ensure normothermia, a temperature of 42 °C was required at the heating element outlet port. The circulation device has built in sensors and monitors to regulate and monitor blood flow, withdrawal pressure, infusion pressure, circuit and patient temperatures, hemofiltration parameters, and has also data storage capacity.

The third phase, the hemofiltration phase, follows perfusion, was characterized by deflation of the catheter balloons and pneumatic cuffs and the restoration of normal circulation. Hemofiltration was then administered for 60 min via the circuit. Blood flow was increased to 200 ml/min and maintained at the aorta withdrawal site and the temperature at the outlet level of the heating element was reduced to 39 °C. A polyamide hemofilter of 2.1 m^2^ (RanD, Medolla, Italy) surface area was used for filtration, after which catheters were withdrawn and vessels repaired. Following percutaneous protocol, compression hemostasis was applied for ~30 min. Protamine was then injected (200 IU/kg) to reverse the anticoagulant effects of heparin.

### Melphalan regimen and pharmacokinetic study

Melphalan was administered at the dose of 30 mg/m^2^, according to previous reports [16, 19, 20] and hemofiltration was performed in all procedures. Pharmacokinetic studies were performed on 5 patients submitted for surgical procedure and five for the percutaneous procedure. For sample collection and analysis, pelvic (inferior cava vein) and systemic (peripheral vein of the arm) blood samples were obtained during perfusion at 5, 7, 14, 17, and 20 min. At the end of perfusion and following balloon deflation, samples of systemic blood, blood exiting patients via the extracorporeal circuit, and ultrafiltrate at 25, 30, 45, 60, and 80 min time points were also collected (Table 1). All samples were processed and stored immediately. Blood samples were centrifuged at 3000 rpm for 10 min, plasma transferred to capped polypropylene tubes and stored at −20 °C. High-performance liquid chromatography (HPLC) analysis of melphalan concentration was performed within 24 h of collection precisely according to the method Mirkou et al. [21]. For pharmacokinetic studies, non-compartment pharmacokinetic analysis (iv infusion NCA model) of melphalan concentrations, with respect to time, were obtained using PK Solver computer software, written in Visual Basic for Applications (VBA) [22]. For hemofiltration (20 to 80 min), total melphalan removal (TMR) was calculated from the ratio of melphalan concentrations in ultrafiltrate and blood exiting the patient. pH and pO_2_ were also measured in blood samples collected from the extracorporeal circuit at 5, 7, 14, 17, and 20 min, during the isolated perfusion phase, using a blood gas analyzer (ABL800 FLEX, Radiometer, Brønshøj, Denmark).

### Statistical analysis

Statistical comparisons between surgical and percutaneous treatments in pharmacokinetics, biochemical and hemodynamic parameters were performed using the non-parametric Mann–Whitney test with the type I error set at 0.05. Median and IQR have been reported. The statistical software STATA (Version 14) was used in this study.

## Results

Patient groups did not exhibit significant differences in age, weight, height and operative times (Table 2). Patients required a median of 10 days post-surgical recovery (IQR 7 to 10). This was significantly longer (p = 0.043) than the time required for recovery following the percutaneous procedure, with a median of 6 days (IQR 5 to 6).

**Table 2:** Demographic data

Groups	Surgical	Percutaneous	MW (p value)
Gender			
Male	3	2	
Female	2	3	
Age (years), median (IQR)	52 (IQR 51 to 60)	68 (IQR 68 to 72)	0.175
Weight (kg), median (IQR)	72 (IQR 71 to 73)	73 (IQR 72 to 76)	0.209
Height (cm), median (IQR)	169 (IQR 164 to 172)	171 (IQR 169 to 172)	0.461
Histology			
Epithelioid pattern	5	5	
Operative time (min), median (IQR)	132 (IQR 126 to 145)	131 (IQR 130 to 156)	0.602
Median (IQR) duration of hospital stay (days)	10 (IQR 7 to 10)	6 (IQR 5 to 6)	0.043

*MW* Mann–Whitney test

Table 3 reports median and IQR blood flow values, withdrawal pressures, infusion pressures, rectal and esophageal temperatures registered during perfusion. The sample included 10 different patients, five submitted for the surgical procedure and five for the percutaneous procedure. Median blood flow values were not significantly different between the 2 groups (p = 0.754), and approximated to 100 ml/min. The two techniques did not show other statistically significant differences (Table 3).

**Table 3:** Comparison of the two techniques during the IPP (20 min) based on hemodynamic and biophysical parameters

Technique	Surgical		Percutaneous		MW
	Median	IQR	Median	IQR	p value
Blood flow (ml/min)	112.5	(111.5 to 120.5)	120	(103.5 to 124.5)	0.754
Withdrawal pressure (mmHg)	−20	(−25 to −8)	−37	(−98 to −35)	0.076
Infusion pressure (mmHg)	101	(94 to 111.5)	114	(109.5 to 120)	0.251
Rectal temperature (°C)	37.5	(36.8 to 37.7)	37.5	(37 to 37.8)	0.602
Esophageal temperature (°C)	36	(35.5 to 36.2)	36.5	(35.7 to 36.4)	0.462

*MW* Mann–Whitney test

In the pharmacokinetic study, in order to compare surgical and percutaneous procedures, the ratios between the area under the plasma concentrations time curve (AUC_0 to 20_ corresponding to the hypoxic perfusion time) of the pelvic versus systemic compartments were the main descriptive parameters considered. The AUC was estimated using the linear trapezoidal method, whereas the maximum plasma concentration (C_max_) was taken as the maximum drug concentration (Table 4). Table 4 also reports median pH and pO_2_ values. Pharmacokinetic parameters were also calculated in peripheral blood samples (Table 5) as: the volume of distribution (Vd); elimination half-time (T½); and total drug clearance (Cl). The median and IQR percentage TMR values in blood exiting patients during hemofiltration (20 to 80 min) were 26.8 (IQR 25.2 to 28.1) in the 5 surgical patients and 28.3 (IQR 26.9 to 29) in the 5 patients subjected to the percutaneous procedure, which were not significantly different (p = 0.465). Therefore, based on data reported in Tables 4, 5, the surgical and percutaneous procedures did not exhibit significant differences.

**Table 4:** Comparison of the two techniques during the IPP (20 min) based on pharmacokinetic and biochemical parameters

Technique	Surgical		Percutaneous		MW
	Median	IQR	Median	IQR	p value
C_max_ perfused compartment (µg/ml)	8.8	(7.3 to 8.8)	7.4	(5.6 to 7.8)	0.346
C_max_ perfused compartment/C_max_ peripheric compartment	7.8	(6.2 to 8.4)	6.6	(5 to 7.5)	0.602
AUC_0 to 20_ perfused compartment/AUC_0 to 20_ peripheric compartment	7.9	(7.2 to 9.9)	5	(4 to 7.9)	0.209
pH extracorporeal circuit	7.39	(7.38 to 7.39)	7.38	(7.38 to 7.39)	0.656
pO_2_ extracorporeal circuit (mmHg)	29.41	(28.56 to 29.64)	29.64	(29.51 to 29.67)	0.248

*MW* Mann–Whitney test, *C*_*max*_ maximum plasma concentration, *AUC*_*0 to 20*_ area under the plasma concentration curve (0 to 20 min)

**Table 5:** Comparison of the two techniques during IPP (20 min) plus hemofiltration (60 min) based on peripheral pharmacokinetic parameters

Technique	Surgical		Percutaneous		MW
	Median	IQR	Median	IQR	p value
C_max_ (µg/ml)	2.3	(2 to 2.5)	2.0	(1.9 to 2)	0.084
AUC_0 to 80_ (µg/ml*min)	81.9	(78.4 to 84.7)	70.3	(64.8 to 81.7)	0.251
T_1/2_ (min)	13	(11.5 to 14)	16	(14 to 16.5)	0.076
Vd [mg/(µg/ml)]	6.5	(5.7 to 6.9)	8.6	(7.7 to 10.2)	0.075
Cl [mg/(µg/ml)/min]	12.3	(11.8 to 13.9)	15.8	(13.8 to 15.9)	0.175

*MW* Mann–Whitney test, *AUC*_*0 to 80*_ area under the plasma concentration curve (0 to 80 min), *T*_*1/2*_ half-life of elimination phase, *Vd* volume of distribution, *Cl* total clearance (extracorporeal plus systemic)

### Tolerability and procedure-related complications

During the 10 procedures, no technical, hemodynamic, or vascular complications were encountered and femoral vessel cannulation was always achieved. Two cases of seroma and 1 case of lymphorrhagia were observed following the surgical procedure (associated with lymphadenectomy in 3 cases) and 1 case of inguinal hematoma was observed following the percutaneous procedure.

### HypoxicIPP with hemofiltration-related toxicity

Two cases of grade 1 neutropenia were detected following the surgical procedure, according to the World Health Organization (WHO) criteria [23] and 1 case of grade 3 neutropenia was observed following the percutaneous procedure. Granulocyte colony-factor supportive therapy was administered to these patients.

Gastro-intestinal toxicity (grade 1 to 2), characterized by transient nausea, was observed in one surgical and two percutaneous patients. Local toxicity (scrotum edema and pain) was detected in 1 patient submitted for inguinal lymphadenectomy.

## Discussion

IPP can be achieved by both surgical or percutaneous approaches. In the present study, we have compared the two methods in terms of pelvic drug concentrations. Pelvic melphalan concentrations were assessed in pelvic venous and systemic blood in order to evaluate differences in pelvic and systemic drug bio-availability during the perfusion phase with vascular block. Melphalan concentrations in systemic blood and ultrafiltrates were also assessed to determine the rate of drug eliminated during hemofiltration. A reliable comparison between surgical and percutaneous procedures was made only when the perfusion phase with extracorporeal blood circulation was exactly the same, with respect to blood flow.

The results obtained from our study show that the two methods did not significantly differ with respect to melphalan bio-availability in the perfused pelvic compartment (AUC_0 to 20_ ratios). The median 5 (IQR 4 to 7.9) and mean 6.42 (SD = 2.56) values for pelvic versus systemic melphalan AUC_0 to 20_ ratio in the 5 percutaneous IPP patients, were lower than those reported by Bonvalot et al. (mean ± SD = 14.1 ± 6) [13], for different doses, sampling times and leakage rates. The percutaneous approach had, however, the clinical advantage of a significantly lower hospitalization time, with earlier (4 days) discharge from hospital, illustrating a further advantage of the percutaneous procedure in terms of cost-effectiveness.

No significant differences in the two methods were observed with respect to tolerability, procedure-related complications and side-effects, and we confirm the data reported by van Ijken et al. [9] concerning the absence of left colon ischemia during hypoxic pelvic perfusion. We stress, however, the importance of using the surgical approach in association with lymphadenectomy.

Due to the particular structure of the percutaneous material (Fig. 2), a higher aspiration pressure was generally required when compared to surgical procedure. This difference in aspiration pressure was constantly adjusted by the equipment in order to guarantee the same mean blood flow value in the extracorporeal circuit. Furthermore, instead of a combination of devices, a single integrated apparatus was used in this study to control perfusion and hemofiltration, as required by European safety legislation (93/42 EU directive).

The rationale behind the 20-min perfusion time used in our study, was based upon in vitro and in vivo studies clearly demonstrating that the cellular uptake of melphalan is rapid and reaches a plateau with 10 min, indicating rapid saturation [20]. Perfusion was performed under conditions of hypoxia, which significantly enhances the efficacy of melphalan [19, 20, 24], with short-term hypoxia also lowering the risk of neo-angiogenesis [25]. Furthermore, both in vitro and in vivo studies have demonstrated that hypoxia-induced acidosis enhances the cytotoxic effects of melphalan by 2.5 to 3.5-fold, probably by increasing cellular drug uptake [19, 20].

Slow flow perfusion was performed in order to reduce drug leakage and lower systemic toxicity, and an important relationship between flow and pressure was observed in the circuit [4]. Aortic pressure falls to 20 mmHg following occlusion and progressively increases with the flow rate. At a flow rate of 100 ml/min, pressure in the pelvic circuit approximates 40 mmHg, whereas at a flow rate of 350 ml/min, pressure increases to 60 mmHg. The pelvic circulation is rich in collaterals, making complete isolation of the vasculature impossible, and increasing perfusion pressure increases drug leakage to the systemic circulation. Therefore, hemofiltration was performed as the protective measure over alternative methods [13, 15, 26] immediately post pelvic perfusion, in order to lower the risk of systemic side effects. Median TMR in the venous blood during the hemofiltration (20 to 80 min) was approximately 27.5%.

## Conclusions

No final conclusions can derive from this pilot study. In terms of tumor exposure to drug, the percutaneous approach for IPP did not statistically differ to the surgical procedure (p = 0.209) at a perfusion blood flow rate of approximately 100 to 120 ml/min. However, our preliminary results, concerning the pharmacokinetics variables, will be invaluable in the future planning of an 80 to 90% statistical power study. According to the ratio of the areas under the melphalan plasma concentration curves in pelvic and systemic compartments during the perfusion (AUC_0 to 20_), the total sample size should be adjusted to 60 patients, given a 90% power and α-level of 5%, and a future avenue of this research topic would be the further miniaturization of materials utilized for the percutaneous procedure.

## Additional file

**Additional file 1.** All data detected are in additional file.

## Abbreviations

IPP: isolated pelvic perfusion
AUC: area under the curve
SD: standard deviation
Fr.: French
CT: computed tomography
HPLC: high-performance liquid chromatography
TMR: total melphalan removal
C_max_: maximum plasma concentration
Vd: volume of distribution
T½: half-time of elimination
Cl: clearance
WHO: World Health Organization
MW: Mann–Whitney test
VBA: Visual Basic for Applications
